# Appropriateness of Cardiovascular Imaging in the Initial Assessment of Possible Acute Coronary Syndrome in the Emergency Department

**DOI:** 10.31083/j.rcm2309293

**Published:** 2022-08-24

**Authors:** Shilpa Vijayakumar, Nishant R. Shah

**Affiliations:** ^1^Division of Cardiology, Department of Medicine, Alpert Medical School of Brown University, Providence, RI 02903, USA

**Keywords:** cardiovascular imaging, acute coronary syndrome, emergency department, risk stratification

## Abstract

Patients presenting with chest pain and related symptoms account for over 6 million emergency department (ED) visits in the United States annually. However, less than 5% of these patients are ultimately diagnosed with acute coronary syndrome (ACS). ED clinicians face the diagnostic challenge of promptly identifying and treating these high-risk patients amidst the overwhelming majority of lower-risk patients for whom further testing and/or treatment is either unnecessary or non-urgent. To assist with and expedite risk stratification and decision-making in this challenging clinical scenario, diagnostic tools like clinical risk scores and high-sensitivity serum biomarkers have been incorporated into care algorithms within the ED. In this narrative review, we discuss how these tools impact the appropriate use of cardiovascular imaging in the initial assessment of patients presenting to the ED with possible ACS.

## 1. Introduction 

Patients presenting with chest pain and related symptoms account for over 6 million emergency department (ED) visits in the United States annually [1]. However, less than 5% of these patients are ultimately diagnosed with acute coronary syndrome (ACS) [2]. ED clinicians face the diagnostic challenge of promptly identifying and treating these high-risk patients amidst the overwhelming majority of lower-risk patients for whom further testing and/or treatment is either unnecessary or non-urgent. To assist with and expedite risk stratification and decision-making in this challenging clinical scenario, diagnostic tools like clinical risk scores and high-sensitivity serum biomarkers have been incorporated into care algorithms within the ED. In this narrative review, we discuss how these tools impact the appropriate use of cardiovascular imaging in the initial assessment of patients presenting to the ED with possible ACS.

## 2. Cardiovascular Risk Stratification 

Evaluation of a patient presenting to the ED with chest pain or other symptoms potentially consistent with ACS should begin with a thorough history and physical examination, a 12-lead electrocardiogram (ECG) within 10 minutes, a blood draw to measure cardiac biomarkers indicative of myocardial injury, and a chest radiograph to evaluate for cardiac or pulmonary causes of symptoms. In addition, a focused bedside ultrasound can be used to promptly evaluate cardiac chamber sizes, global and regional left ventricular function, cardiac valvular pathology, and to assess for any intracardiac masses or pericardial effusion [3]. Since focused cardiac ultrasounds are being increasingly used by ED physicians and other clinicians to guide medical decision making, it is recommended that all systems used for focused ultrasounds have a method of recording and archiving data for later review [4]. Anginal symptoms are generally recognized as retrosternal or left-sided chest discomfort that gradually builds in intensity over minutes and are often triggered by physical exercise or emotional stress. Chest pain that occurs at rest or with minimal exertion may be indicative of ACS. Relief of chest pain with nitroglycerin is not a specific indication of myocardial ischemia and should not be used as a diagnostic measure [3].

The ultimate goals of evaluation of chest pain in the ED are to identify life-threatening causes of chest pain (i.e., ACS, acute aortic syndromes, pulmonary embolism, esophageal rupture), ascertain hemodynamic and clinical stability, and determine whether further inpatient evaluation and hospitalization are warranted or if the patient can safely be managed in the outpatient setting. In cases of possible ACS, a clinical decision pathway allows for the classification of patients into high, low, or intermediate probability of ACS. Beyond the diagnostic utility of expedited identification of ACS, tools such as risk scores and high-sensitivity cardiac biomarkers can also estimate the probability of future adverse clinical events [3].

### 2.1 High-Risk

Patients with a high likelihood of ACS include those with angina or anginal-equivalent symptoms, ECG changes suggestive of myocardial injury and/or ischemia, and elevated or rising serum cardiac troponin levels [5]. Amongst these patients, those with the highest risk of major adverse cardiovascular events (MACE) include patients with ST-segment elevation myocardial infarction (STEMI) and non-ST-segment elevation ACS (NSTE-ACS) with high-risk features including refractory angina, signs or symptoms of heart failure (HF), hemodynamic instability, ventricular arrhythmias (sustained ventricular tachycardia or ventricular fibrillation), and mechanical complications such as acute mitral regurgitation [6]. These patients should undergo invasive coronary angiography within 2 hours of arrival in the ED.

Multiple randomized controlled trials and meta-analyses have validated the benefit of early invasive coronary angiography in high-risk patients with NSTE-ACS [7, 8, 9, 10, 11, 12, 13]. Thus, in patients with NSTE-ACS who do not fall into the above highest-risk profile but still have an elevated risk for clinical events, an early invasive strategy with coronary angiography and intervention, if indicated, is warranted [6]. Coronary angiography within 24 hours is generally recommended in patients who fall into any high-risk category including an established non-ST-segment elevation myocardial infarction (NSTEMI) by defined by significant elevation and/or rise in cardiac troponin, new or presumably new contiguous ST-segment depressions, and clinical risk scores indicative of high-risk (e.g., Global Registry of Acute Coronary Events (GRACE) score >140) [6]. The 2020 European Society of Cardiology guidelines also recommend early invasive approach with coronary angiography within 24 hours in patients with resuscitated cardiac arrest without ST-segment elevation or cardiogenic shock [14]. Current 2014 American College of Cardiology/American Heart Association guidelines also advise invasive coronary angiography within 25 to 72 hours in patients with reduced left ventricular systolic function (LVEF) <40%, early post-infarction angina, diabetes mellitus, percutaneous coronary intervention (PCI) within 6 months, prior coronary artery bypass graft (CABG), and/or clinical risk scores suggestive of intermediate-to-high risk (GRACE risk score 109–140 or Thrombolysis in Myocardial Infarction (TIMI) score ≥2) [6].

As mentioned above, risk prediction models in patients with ACS have been developed to approximate risk of adverse events including death or myocardial infarction. These models can guide management strategies. Two of the most commonly used scores include the GRACE risk score and the TIMI risk score. GRACE uses eight variables to predict risk: age, heart rate at admission, systolic blood pressure at admission, serum creatinine, cardiac arrest at admission, ST-segment deviation on ECG, elevated initial cardiac biomarkers, and Killip classification for heart failure [15, 16, 17]. In patients with NSTE-ACS, using the endpoint of in-hospital mortality, a GRACE risk score of 1–108 represents low risk and confers <1% probability of death, a score of 109–140 represents intermediate risk and confers 1–3% probability of death, and a score of 141–372 represents high risk and confers >3% probability of death [15, 18]. The TIMI risk score predicts 14-day risk of mortality, new or recurrent myocardial infarction, or severe recurrent ischemia requiring urgent revascularization using seven variables: age 65 or greater, at least 3 risk factors for coronary artery disease (CAD) (hypertension, hypercholesterolemia, diabetes, family history of CAD, active smoking status), prior coronary stenosis of 50% or greater, ST-segment deviation on ECG, at least 2 anginal events within the prior 24 hours, use of aspirin within the prior 7 days, and elevated serum cardiac markers. TIMI scores of 0–2 represent low risk, 3–4 represent intermediate risk and 5–7 represent high risk. This risk score has been validated by numerous successive studies in patients presenting to the ED with chest pain [19, 20, 21, 22].

### 2.2 Low-Risk 

Providers in the emergency room also face the challenge of identifying low-risk patients who can be safely discharged without any additional testing or hospitalization. Low-risk chest pain is currently defined as having <1% 30-day risk of MACE [23, 24]. A survey of 1029 ED clinicians revealed 41% accepted a MACE risk of <1% and the majority (56.8%) accepted a MACE risk of ≤0.5% in patients being discharged home [25]. High-sensitivity troponin assays and clinical risk stratification tools can be used to identify this low-risk cohort in a timely manner.

#### 2.2.1 High-Sensitivity Cardiac Troponin-Based Stratification 

High-sensitivity cardiac troponin (hs-cTn) assays allow for the detection of very low concentrations of hs-cTn with great precision, facilitating expedited triage and rule-out of myocardial infarction. Use of hs-cTn can identify low-risk patients either upon presentation or by the use of a 0/1-hour or 0/2-hour algorithm.

*Upon Presentation*: In patients whose onset of chest pain was at least 3 hours prior to presentation to the ED, an initial hs-cTn below the assay detection limit (typically defined at <5 ng/L) can safely and effectively exclude acute myocardial infarction (AMI). Hs-cTn has high negative predictive value, and patients with undetectable initial hs-cTn have low rates of MACE, even up to 1 year after discharge [26, 27, 28, 29, 30].

*0/1-Hour or 0/2-Hour Algorithms*: Recently, various algorithms have been developed for prompt rule-out of AMI using hs-cTn levels at presentation and either 1 or 2 hours after arrival. These protocols have been studied in patients with varying onset of chest pain (including those with onset <3 hours prior to presentation). AMI can be ruled out in patients in whom the initial hs-cTn as well as the 1-hour or 2-hour delta hs-cTn are below the low thresholds for the assay. Patients who rule out by these algorithms can be safely discharged without any further risk stratification tools or testing [31, 32, 33, 34].

Patients who do not rule out at presentation or within 1 to 2 hours of arrival will require a 3-hour hs-cTn value and may benefit from further risk stratification by a clinical risk score or other testing modalities [35].

#### 2.2.2 Clinical Pathways of Risk Stratification

Clinical risk scores allow for the integration of symptoms, risk factors, physical examination, ECG findings, and cardiac biomarker abnormalities to effectively risk stratify patients presenting to the ED with chest pain. These scores can be used adjunctively to hs-cTn in patients with initial and serial cTn or hs-cTn assay <99th percentile who do not rule out by hs-cTn upon initial presentation or within one hour. The clinical pathways with strongest evidence for safely identifying low-risk patients include the TIMI score, the History, Electrocardiogram, Age, Risk Factors and Troponins (HEART) score, and the Emergency Department Assessment of Chest Pain Score (EDACS) [36, 37]. The No Objective Testing Rule (NOTR) has also been developed to identify patients who do not require further objective testing for CAD and has been externally validated [38, 39, 40].

*TIMI/ADAPT*: The 2-Hour Accelerated Diagnostic Protocol to Assess Patients with Chest Pain Symptoms Using Contemporary Troponins (ADAPT) trial assessed an accelerated diagnostic pathway using TIMI score, ECG findings, and initial and 2-hour cTn assays. In patients with a TIMI score of 0, no ischemic ECG changes, and initial and serial 2-hour cTn assay <99th percentile, ADAPT diagnostic pathway had a negative predictive value of 99.7% in identifying patients at low 30-day risk of MACE [41]. A validation study modified this diagnostic pathway to incorporate patients with TIMI scores of 0 and 1 and found no difference in 30-day MACE [42].

*HEART*: Unlike GRACE and TIMI scores mentioned previously, the HEART pathway is an accelerated diagnostic protocol specifically designed to risk stratify patients with chest pain of unclear etiology and identify patients at low risk for MACE. The HEART score incorporates history, ECG findings, age, risk factors, and troponin [43]. A HEART score of ≤3 and initial/serial hs-cTn assay <99th percentile over 3 hours has been found to be associated with a 30-day MACE rate of 0.4% [35]. Studies comparing HEART, TIMI, and GRACE scores showed the HEART score was superior to TIMI and GRACE scores in estimating 30-day MACE and identifying low-risk patients [44, 45].

*EDACS*: EDACS pathway is an emergency-medicine derived protocol that combines EDACS score with ECG findings and cTn initially and at 2-hours to identify patients at low-risk who can be safely discharged from the ED. EDACS incorporates age, sex, history of CAD or risk factors for CAD, and symptoms (including diaphoresis, pain that radiates to arm/shoulder/neck/jaw, pain occurring with or worsened by inspiration, and pain reproduced by palpation) [46]. An EDACS score <16 in combination with no new ischemic findings on ECG and initial and serial 2-hour cTn assay <99th percentile signifies low risk with <1% 30-day MACE risk. The EDACS diagnostic protocol has been validated by numerous subsequent studies [47, 48, 49]. One retrospective study suggested improved accuracy in prediction of 60-day MACE using EDACS protocol compared to the HEART pathway, but no prospective trials have compared the two scores [50].

*NOTR*: In patients without ECG abnormalities and initial and serial cTn assay <99th percentile, NOTR score was developed to recognize patients who do not require any further testing. The NOTR tool incorporates age, history of myocardial infarction, and risk factors for CAD. A score of 0 correlates with low risk of 30-day MACE rate [38, 39, 40]. Although NOTR criteria and HEART criteria are similar, NOTR has more rigorous standards for defining low risk. Thus, NOTR has high sensitivity for estimating 30-day MACE rate, but the HEART score may outperform NOTR in identifying more patients who are suitable for early discharge [38].

In patients who clearly fall into the categories of low-risk and high-risk as detailed above, performing additional cardiovascular imaging is rarely appropriate. In most patients in the emergency-based chest pain unit, routine functional testing provides no significant benefit [51]. Particularly in the current era of hs-cTn assays, after negative hs-cTn results at 0 and 90 minutes, and after ED clinical assessment of low-risk, objective cardiac testing offered little therapeutic yield and did not improve prediction for 30-day acute myocardial infarction or revascularization [52]. In all other patients, including those at low-to-intermediate risk, intermediate risk, and intermediate-to-high risk, the use of cardiovascular imaging for further assessment and risk stratification is appropriate. Intermediate-risk patients do not have overt evidence of myocardial injury by troponin, although some may have chronic/minimal elevations. Cardiac testing for these patients is often performed in the inpatient setting via hospitalization or in a dedicated observation unit. Various imaging techniques for functional and anatomic assessment and the appropriateness of each technique will be discussed below.

## 3. Functional Assessment 

In patients in whom additional testing is appropriate, functional testing can provide valuable hemodynamic data and allow for further risk stratification. In patients without significant baseline ECG abnormalities who are able to exercise to an adequate workload, the simplest and most widely available method of functional testing is exercise ECG stress testing. Exercise ECG stress testing can not only provoke ECG abnormalities indicative of myocardial ischemia, but also provide useful prognostic information including exercise capacity, heart rate response and recovery, and blood pressure response (including the presence of exercise-induced hypotension) [53, 54]. Contraindications to exercise EGG testing in the ED setting include acute myocardial infarction, ongoing unstable angina, uncontrolled cardiac arrhythmias, symptomatic severe aortic stenosis, decompensated heart failure, active endocarditis, acute pulmonary embolism or deep vein thrombosis, acute myocarditis or pericarditis, acute aortic dissection, significant resting hypertension, and inability to exercise [55, 56]. ECG stress testing has been used to practically exclude ACS in low-risk populations [53, 57]. However, studies also report lower diagnostic accuracy, increased rates of indeterminate testing, and higher rates of false positive tests with exercise ECG testing [58, 59]. This often leads to additional testing and higher costs. Furthermore, with the increased utilization of hs-cTn and clinical decision scoring, exercise stress testing does not significantly contribute to clinical decision making in low-risk patients [60]. To enhance diagnostic accuracy and risk stratification of intermediate-risk populations, cardiovascular imaging is often incorporated into functional assessment.

### 3.1 Echocardiography 

#### Stress Echocardiography 

Immediate stress echocardiography has been shown to be equally as safe and more efficient in the triage and risk stratification of low-risk chest pain patients in the ED when compared to admission to an observation unit [61]. Several head-to-head comparisons of stress echocardiography to other modalities have also shown promising results. Compared to exercise ECG stress testing, exercise stress echocardiography provided greater diagnostic accuracy and prognostic yield. Stress echocardiography also resulted in fewer referrals for further diagnostic investigation including coronary angiography and was consequently more cost-effective during short- and long-term follow-up [62, 63]. Dobutamine stress echocardiography was also feasible, safe, and cost-effective when compared to exercise ECG stress in the triage of low-risk patients with chest pain in the ED [64]. Compared to exercise myocardial single-photon emission computed tomography (SPECT) in approximately 500 patients evaluated in a chest pain unit, exercise stress echocardiography performed equally well in diagnosing CAD and in short-term prognostication. Exercise stress echocardiography also demonstrated higher positive predictive value and led to fewer unnecessary invasive coronary angiograms [65]. In a recent single-center study comparing early ED use of coronary computed tomography angiography (CCTA) and stress echocardiography (including dobutamine stress if appropriate), 400 low-to-intermediate risk, predominantly ethnic minority patients with no known CAD and negative initial serum troponin level were randomized to either immediate CCTA or stress echocardiography. Compared with CCTA, stress echocardiography resulted in fewer hospitalizations, reduced length of stay, decreased radiation exposure, and improved patient satisfaction. There was no difference between the two groups in safety outcomes or downstream resource utilization including subsequent ED visits, cardiology outpatient visits, and primary care outpatient visits [66]. The use of ultrasound-enhancing agents can further improve diagnostic accuracy and are recommended when two or more contiguous segments of the left ventricle are inadequately visualized [67]. Contraindications to stress echocardiography include uncontrolled heart failure or respiratory failure, high-risk angina or active ACS, ventricular arrhythmias, poor acoustic windows, severe systemic hypertension, and any contraindication to dobutamine if pharmacologic stress is needed [3]. As opposed to other modalities, stress echocardiography is free of ionizing radiation, readily available at most hospitals, cost-effective, and can provide adjunctive information on valvular function and diastolic function. An important limitation of stress echocardiography is limited visualization and/or poor image quality in certain populations including obese patients, patient with tachycardia, and those with significant lung disease.

### 3.2 Radionuclide Myocardial Perfusion Imaging

Myocardial perfusion imaging (MPI) is safe, efficacious, and cost-effective when utilized in the ED setting [68, 69]. Resting MPI was evaluated in a multicenter randomized control trial to assess if it offered benefit in an ED evaluation strategy for low-to-intermediate risk patients with suspected myocardial ischemia but no initial ECG changes indicative of ischemia. In this study of over 2000 patients, incorporation of resting MPI into an accelerated ED diagnostic protocol resulted in reduced rates of unnecessary hospitalization without any increased 30-day rates of adverse outcomes but did not result in faster discharge times from the ED [70]. These results have been subsequently reproduced [71]. Stress MPI has also been shown to offer incremental benefit when added to an ED evaluation protocol. When compared to standard evaluation strategy of clinical assessment, addition of stress MPI in the evaluation strategy had a significantly lower admission rate with no significant difference in 30-day or 1-year outcomes between the stress MPI and clinical assessment groups [72]. In addition, a recent single-center retrospective cohort study of 213 patients referred for vasodilator or exercise rest-stress MPI with mildly abnormal hs-cTn values (but not high risk as defined in this study by hs-cTn ≥52 ng/L or a one-hour change of ≥5 ng/L) and a non-ischemic resting ECG found that rest-stress MPI appears safe in this population, approximately 15% of patients had evidence of myocardial ischemia demonstrated by rest-stress MPI, and the incidence of myocardial ischemia correlated with higher HEART scores [73]. Patients with mildly abnormal hs-cTn levels were more often male, older, and had pre-existing CAD. These patient characteristics were also seen in a larger, multi-center diagnostic study [74]. Thus, functional testing with MPI may be particularly beneficial in this patient cohort. Contraindications to nuclear stress testing include ACS or high-risk angina features, severe systemic hypertension and contraindications to vasodilator administration including significant arrhythmias, hypotension, bronchospastic disease, recent use of dipyridamole and use of caffeine within 12 hours. Limitations of nuclear imaging include limited nuclear laboratory availability, radiation exposure, and artifacts caused by motion, attenuation, or extracardiac activity [75].

### 3.3 Cardiovascular Magnetic Resonance Imaging 

In patients presenting to the ED with chest pain in whom cardiac biomarkers were negative, adenosine stress CMR has both high sensitivity and specificity in predicting significant CAD during 1-year follow-up [76]. Stress CMR in the ED setting has been investigated by a series of small, single-center, randomized control trials. The first of these trials included intermediate-to-high risk patients based on a TIMI score of 2 or greater (but with negative ECG and cardiac biomarkers) and those with known CAD. When compared to standard inpatient care, the incorporation of stress CMR in an observation unit strategy resulted in lower median cost without any missed cases of ACS [77]. Long-term healthcare expenditures were also evaluated, and a stress CMR strategy in an observation unit reduced cumulative costs at 1-year follow-up [78]. Among lower-risk patients, a study comparing a mandatory stress CMR strategy to a stress testing modality selected by patients’ clinicians (often stress echocardiography or radionuclide myocardial perfusion imaging) found no differences between the groups in length of stay or 30-day incidence of ACS. However, when compared with a mandated stress CMR, the ability of a physician to select testing modality was more cost-effective [79]. The same authors subsequently randomized patients to either a stress CMR and observation unit protocol or usual care in the ED observation unit followed by consultation with cardiologists and/or internists regarding hospitalization versus discharge. The stress CMR group had reduced rates of coronary artery revascularization, hospital readmission, and repeat cardiac testing without an increase in the incidence of ACS at 90 days [80]. When compared directly to stress echocardiography in intermediate-risk patients, stress CMR performed within 12 hours of presentation was equally as safe and was a stronger predictor of significant CAD [81]. A recent small, randomized control trial compared a CMR or CCTA strategy with routine clinical care in 207 patients presenting to the ED with acute chest pain with a type 1 myocardial infarction by elevated hs-cTn levels (>14 ng/L) and inconclusive ECG findings. The median hs-cTn level in the study was 78 ng/L. A CMR- or CTA-first strategy reduced referral for invasive coronary angiography during initial hospitalization and at 1-year follow-up. There was a low event rate overall in the study but a trend towards decreased MACE and reduced complications in the CMR- and CTA-first arm. A stress CMR strategy did not reduce invasive coronary angiography as much as a CTA strategy but CMR more frequently resulted in a clinically relevant diagnosis including myocarditis, cardiomyopathy, and myocardial infarction in the absence of obstructive CAD [82]. Limitations to stress CMR include limited access, long scanning protocols, necessity for specialized staffing, limited high-resolution anatomic assessment of the coronary arteries, and cost. Contraindications to stress CMR include renal dysfunction (with glomerular filtration rate <30 mL/min/1.73 $m^{2}$), contraindications to vasodilator administration, implanted medical devices which are unsafe for CMR, foreign metal in the body, severe claustrophobia, and caffeine use within 12 hours [3].

## 4. Anatomic Assessment with Coronary Computed Tomography Angiography 

The safety and efficacy of CCTA in patients with acute chest pain has been validated by numerous clinical trials [83]. An early study comparing multi-slice CT with standard of care (including traditional nuclear stress testing) in patients with low-risk acute chest pain found both strategies to be safe and effective at excluding or diagnosing CAD. Multi-slice CT was able to establish a more rapid diagnosis and facilitate earlier discharge from the ED. Limitations in the study included cases of insufficient image quality and cases of coronary lesions of intermediate severity whose physiologic significance remained unclear [84]. Another study, the CT-STAT trial, randomized 699 low-to-intermediate risk patients with acute chest pain to either CCTA or rest-stress MPI. CT-STAT demonstrated that compared to MPI, CCTA resulted in a 54% reduction in time to diagnosis and 38% reduction in ED cost with no differences in MACE at 60 days [85]. A larger trial of 1370 low-to-intermediate risk patients presenting with possible ACS showed that a CCTA-based strategy allowed for a safe and quick discharge from the ED compared to traditional care [86]. ROMICAT II was a large, prospective study which randomized 1000 intermediate-risk, younger patients (age 40–74 years) with acute chest pain and symptoms suggestive of ACS but without ischemic ECG changes or positive troponin value to CCTA versus standard ED care with a primary endpoint of length of hospital stay. The study authors found that a CCTA-strategy had a high negative predictive value and was able to reduce length of stay by 7.6 hours (23 hours in CCTA group versus 31 hours in standard ED care group) with no difference in MACE at 28 days and no missed ACS in either arm [87]. The ACRIN-PA trial also evaluated the safety of a CCTA-based strategy compared to traditional care in 1370 low-to-intermediate risk patients with acute chest pain and demonstrated that CCTA was associated with a higher rate of detection of CAD, shorter length of stay and higher rate of discharge from the ED compared to traditional care with no increase in 30-day MACE in patients with a normal CCTA [86]. Lastly, the CATCH trial compared CCTA with standard care (either bicycle exercise ECG stress test or MPI) in 600 patients with acute-onset chest pain in Denmark. CCTA-based strategy reduced the risk of MACE during the median follow-up period of 18.7 months compared to standard care [88]. A meta-analysis including the trials discussed established that although CCTA was safe and associated with lower costs and length of stay, the use of CCTA was associated with increased downstream use of invasive coronary angiography and revascularization by 2% when compared with usual care with unclear overall benefit on patient outcomes [89]. These findings were corroborated by subsequent randomized trials and meta-analyses [90, 91, 92].

The above studies were all performed prior to the approval and widespread use of hs-cTn. In the era of hs-cTn, a CCTA-based strategy was evaluated by the BEACON trial, a European randomized study evaluating 500 patients with symptoms suggestive of ACS who did not require invasive coronary angiography or have a history of ACS or coronary revascularization. CCTA was found to be safe and associated with less outpatient testing/cost. However, in the era of hs-cTn, BEACON did not show any significant difference between CCTA and standard of care in length of stay or diagnosis of significant CAD requiring revascularization. In addition, the BEACON trial did not show any significant 30-day difference in outcomes between the two groups [93].

A CCTA-first strategy has also been evaluated in intermediate-to-high risk patients with variable results. The VERDICT trial evaluated the outcome of patients with confirmed non-ST elevation acute coronary syndromes (NSTE-ACS) to either very early or standard invasive coronary angiography. There was an additional observational component of clinically blinded CCTA conducted prior to angiography in both groups. VERDICT showed a high diagnostic accuracy of CCTA, with a 96.5% sensitivity and overall accuracy of 88.7% [94]. The RAPID-CTCA trial evaluated CCTA with usual care in 1748 patients with suspected ACS and either prior history of CAD (34%), hs-cTn >99th percentile (57%), or abnormal ECG (61%). CCTA did not result in a reduction of MACE at 1-year in these intermediate-to-high risk patients. Furthermore, CCTA was associated with a modest increase in length of stay and cost [95].

Many centers that perform CCTA in the ED have a protocol that begins with acquisition of gated, non-contrast images to quantify coronary artery calcium (CAC). An accumulating body of evidence, synthesized in a recent meta-analysis [96], suggests that patients with acute chest pain without a history of CAD, ischemic ECG findings, or abnormal serum troponin levels who have a CAC score of zero have less than 1% per year risk of major adverse cardiovascular events. As such, these very low risk patients are highly unlikely to benefit from hospital admission or further diagnostic testing, including completion of the remainder of CCTA protocol. However, as the authors of the meta-analysis acknowledge in their limitations, the 8 prospective studies they included in their analysis enrolled predominantly white patients in the United States and additional studies are needed to understand whether CAC of zero in portends similarly low risk in patients with acute chest pain from other ethnic groups and geographic regions.

Contraindications to CCTA include allergy to iodinated contrast, clinical instability, renal impairment, heart rate variability/arrhythmia, contraindication to beta blockade if elevated heart rate is present, and inability to cooperate with breath-holding instructions [3]. Many hospitals also do not have the capability to perform CCTA around the clock. Additional concerns regarding CCTA include detection of intermediate-severity stenosis of unclear significance requiring further non-invasive functional testing or potentially unnecessary coronary angiography and increased downstream utilization of resources due to incidental findings [3].

## 5. Approach to the Patient Presenting with Possible ACS in the ED

**Fig. 1.**
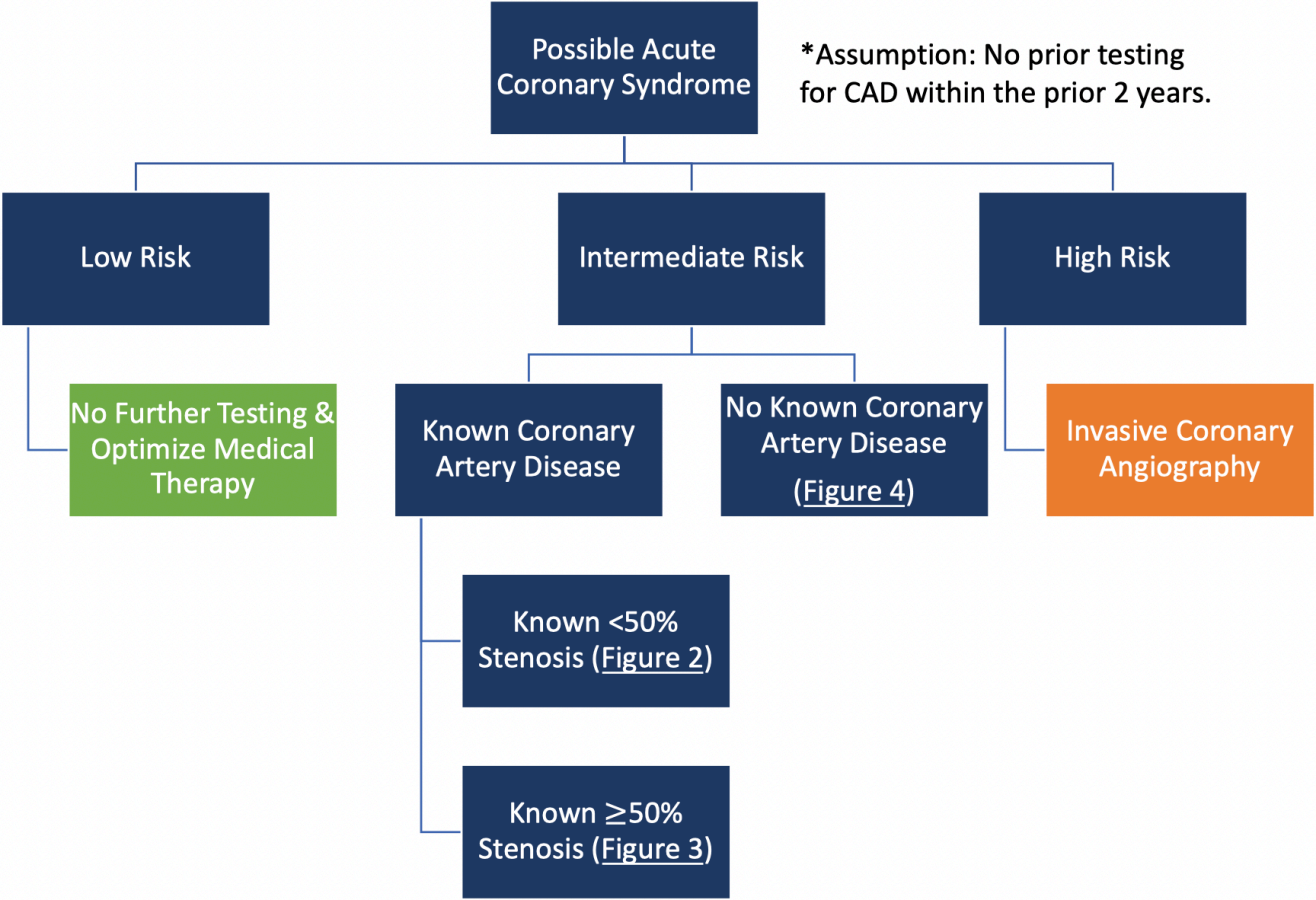
**Risk-based initial decision-making in patients with possible ACS presenting to the ED**.

Clearly our current armamentarium for the assessment of patients presenting with possible ACS in the ED is vast and still expanding. In all patients presenting with possible ACS to the ED, the current guidelines place particular emphasis on a thorough history, physical examination, ECG, and serum hs-cTn. Hs-cTn is the preferred cardiac biomarker since it allows for more accurate detection and exclusion of ACS. The use of clinical decision pathways for further risk stratification are also recommended as part of routine evaluation of all patients with acute chest pain and suspected ACS. In patients at high-risk, invasive coronary angiography is recommended (Fig. 1). In patients at low risk based on clinical decision pathway and hs-cTn, no further cardiac testing is required, and these patients may be safely discharged from the ED (Fig. 1) [3].

**Fig. 2.**
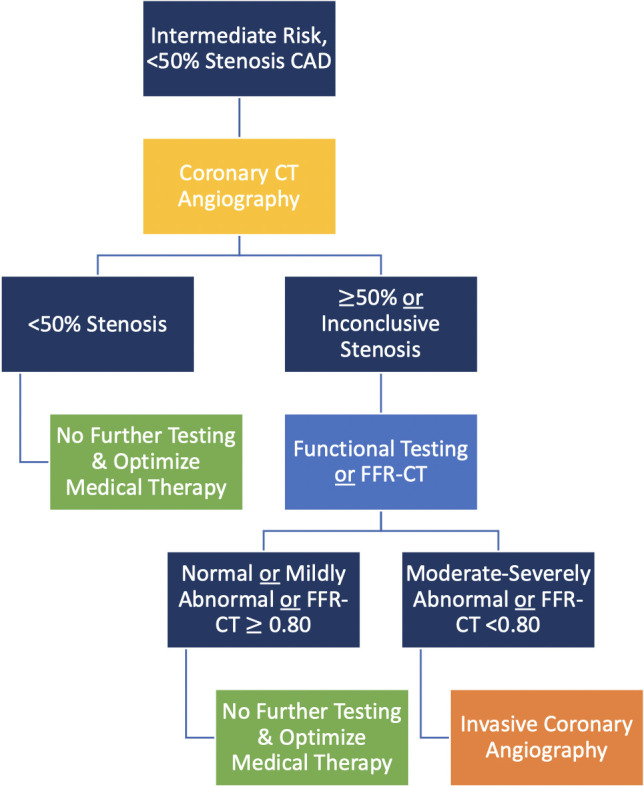
**Coronary CT angiography is the test of choice in intermediate-risk patients with known <50% stenosis CAD on testing performed more than 2 years prior**.

**Fig. 3.**
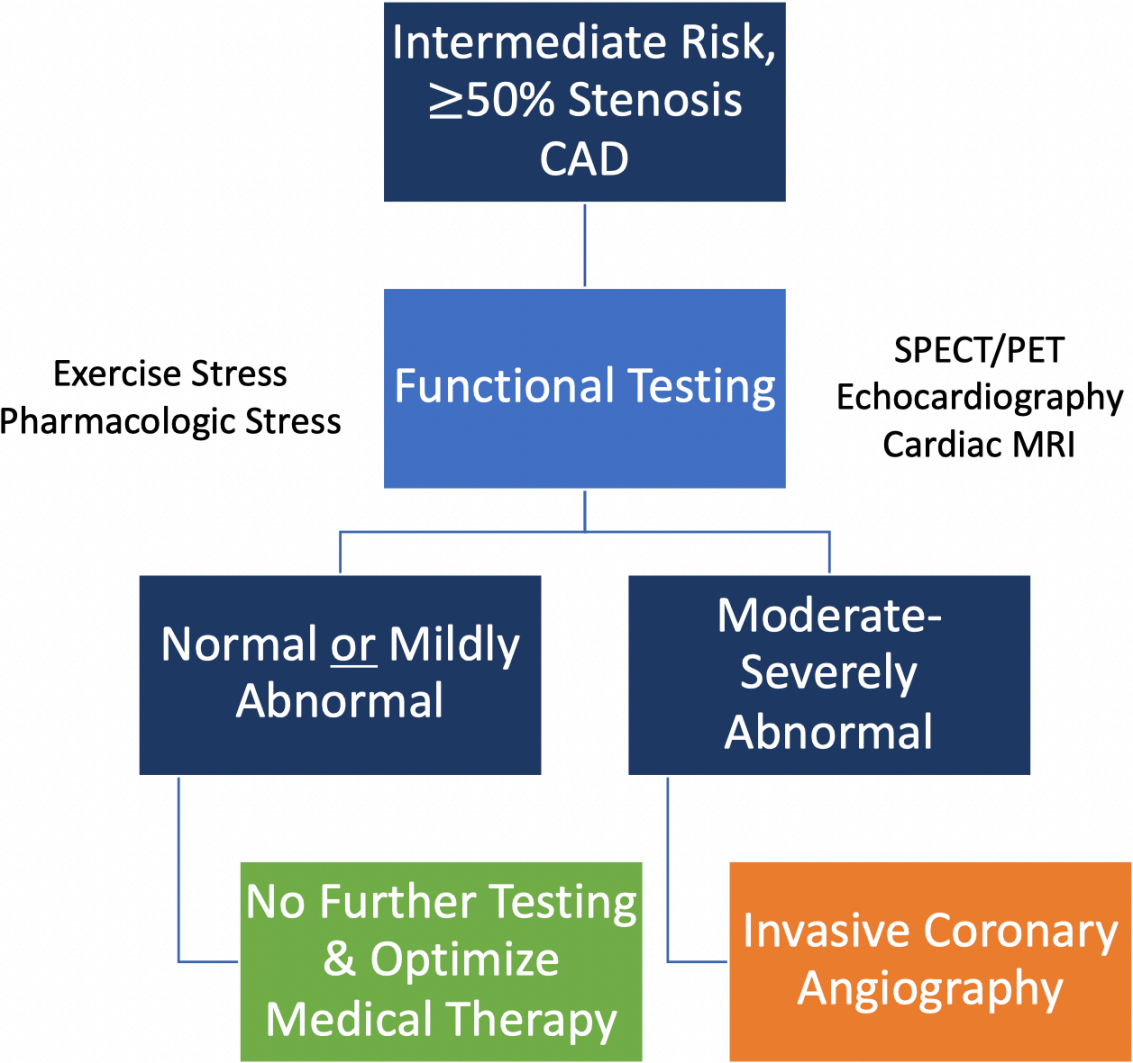
**Functional (stress) testing is preferred in intermediate-risk patients with known ≥ 50% stenosis CAD on testing performed more than 2 years prior**.

**Fig. 4.**
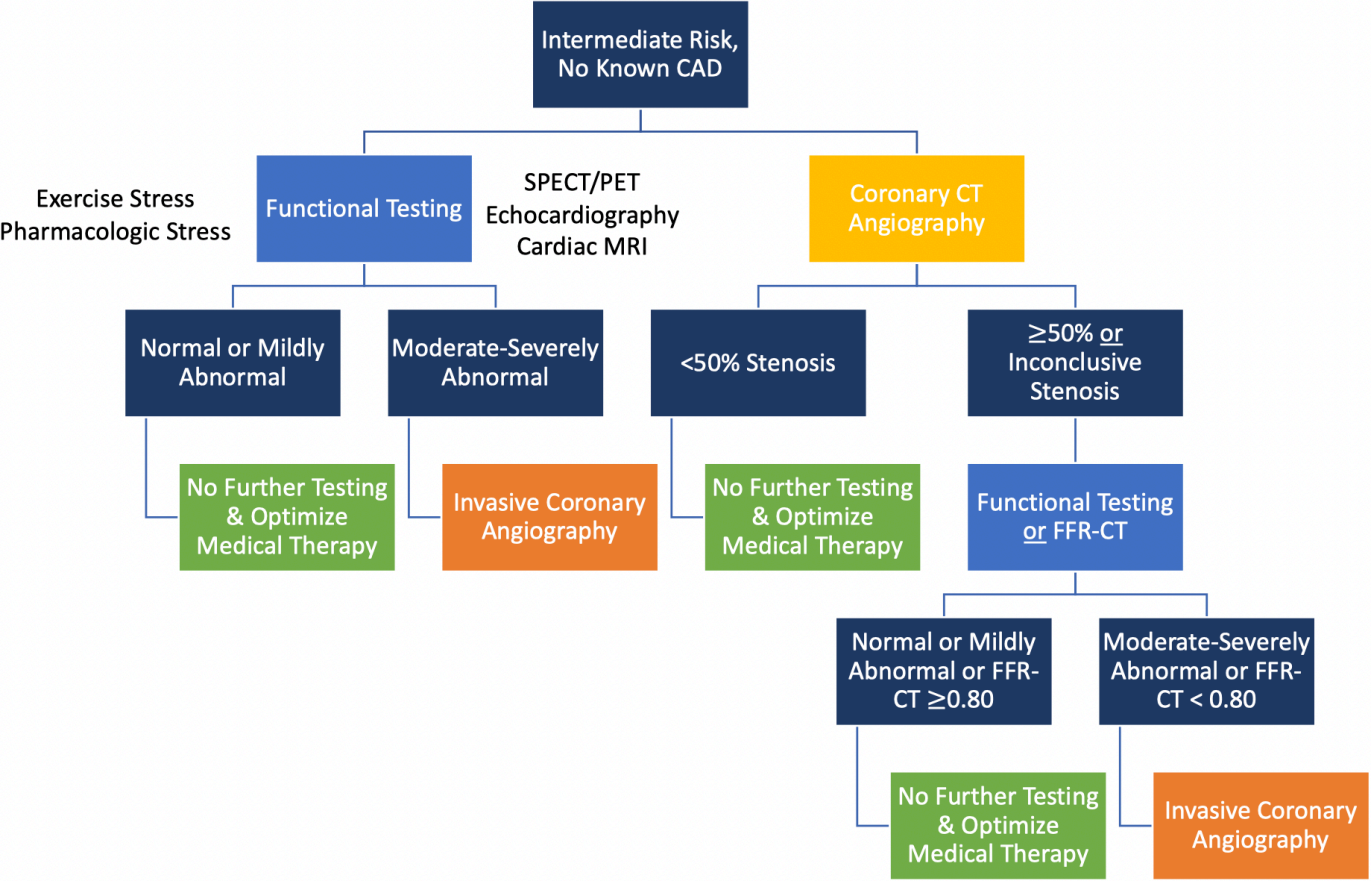
**Coronary CT angiography and functional (stress) testing are both first-line options to evaluate intermediate risk patients with no known CAD**.

Patients who don’t fall clearly into either of these categories, including those at low-to-intermediate risk, intermediate risk, or intermediate-to-high risk may benefit from further diagnostic testing as shown in Figs. 2,3,4.

In intermediate-risk patients with known CAD (Figs. 2,3) [3]:

• Guideline-directed medical therapy should be optimized prior to additional cardiac testing if new or worsening symptoms are present.

• In patients with acute chest pain and known left main, proximal left anterior descending stenosis or multivessel CAD or history of prior coronary revascularization, invasive coronary angiography is recommended. Invasive coronary angiography should also be considered in patients with known CAD presenting with daily or weekly symptoms.

• In patients with known prior non-obstructive CAD, CCTA should be considered to evaluate for progressive CAD.

• For patients with known obstructive CAD without any of the high-risk features mentioned earlier, functional testing is recommended.

In intermediate-risk patients with no known CAD (Fig. 4) [3]:

• If recent testing (either within 1 year for stress testing or within 2 years for CCTA) was normal, no further testing is indicated.

• If a patient underwent a recent inconclusive or mildly abnormal stress test within the past year, CCTA is recommended for exclusion of obstructive CAD.

• If a patient has evidence of known moderate-to-severe ischemia on functional testing within the past year and no prior anatomic testing, invasive coronary angiography is recommended.

• Among patients with no recent testing, additional diagnostic testing can include either functional or anatomic testing with personalized test selection based on several patient- and facility-level factors, including exertional vs. non-exertional symptoms, ability to exercise, presence of a left bundle branch block and/or ventricular pacing, known iodine contrast allergy, known moderate-severe renal dysfunction, and available expertise and/or equipment to perform and interpret specific tests.

In general, CCTA has excellent negative predictive value and will likely have increased value in the ED setting if used on a well-defined, lower-risk population, including patients with no known CAD or mild/non-obstructive CAD. CCTA is also advantageous in patients with known anomalous coronary arteries or in patients in whom further evaluation of the aorta or pulmonary arteries would be useful. In patients with higher atherosclerotic burden, CCTA may overestimate the significance of CAD and is associated with increased downstream testing and procedures. Functional testing can provide insight into the hemodynamic consequences of known CAD and identify flow-limiting CAD with stress. Thus, these tests will be more beneficial in a higher-risk population for risk stratification and ischemia-guided management. If myocardial scar/infarct or coronary microvascular dysfunction is suspected, PET or CMR are particularly valuable at identifying microvascular dysfunction, differentiating ischemia from infarct, and quantifying scar burden.

## 6. Conclusion and Future Directions

Acute chest pain is one of the most common symptoms for which a patient seeks emergency medical care, and the rapid triage and management of these patients is an ongoing challenge faced by ED clinicians. In the era of hs-cTn and validated clinical decision pathways, low-, intermediate-, and high-risk patients are more clearly defined and identified. Patients at low risk of MACE generally do not require any further diagnostic cardiac testing. Patients at high risk should usually proceed directly to invasive coronary angiography for prompt diagnosis and treatment of ACS. Patients who do not clearly fall into either low- or high-risk categories often benefit from further diagnostic imaging in the ED, either anatomic or functional testing. Anatomic testing with CCTA and various functional imaging modalities including stress echocardiography, stress MPI, and stress CMR each offer unique benefits, and randomized trial data scrutinizing each modality have yielded promising results. However, there remain gaps in our understanding of the utilization of these modalities across a spectrum of cardiovascular risk (including those with mildly abnormal hs-cTn) and long-term outcomes. Future randomized trials should focus on assessment of long-term outcomes (including effectiveness, safety, cost, and downstream resource utilization) of an imaging-guided diagnostic strategy. Data from large, real-world studies should also help refine test selection and allow for better integration of various modalities into clinical practice.
